# A Pilot Study of a Natural Food Supplement as New Possible Therapeutic Approach in Chronic Kidney Disease Patients

**DOI:** 10.3390/ph13070148

**Published:** 2020-07-10

**Authors:** Annalisa Noce, Alessio Bocedi, Margherita Campo, Giulia Marrone, Manuela Di Lauro, Giada Cattani, Nicola Di Daniele, Annalisa Romani

**Affiliations:** 1 UOC of Internal Medicine-Center of Hypertension and Nephrology Unit, Department of Systems Medicine, University of Rome Tor Vergata, Via Montpellier 1, 00133 Rome, Italy; giul.marr@gmail.com (G.M.); dilauromanuela@gmail.com (M.D.L.); didaniele@med.uniroma2.it (N.D.D.); 2 Department of Chemical Sciences and Technologies, University of Rome Tor Vergata, Via della Ricerca Scientifica 1, 00133 Rome, Italy; bcdlss01@uniroma2.it (A.B.); giada.cattani@uniroma2.it (G.C.); 3 PHYTOLAB (Pharmaceutical, Cosmetic, Food Supplement, Technology and Analysis)-DiSIA, University of Florence, Via U. Schiff, 6, 50019 Sesto Fiorentino, Italy; margherita.campo@unifi.it; 4 School of Applied Medical, Surgical Sciences, University of Rome Tor Vergata, via Montpellier 1, 00133 Rome, Italy

**Keywords:** inflammatory biomarkers, bioactive compounds, natural antioxidants, vitamin C, conservative therapy, erythrocyte glutathione transferase, human oxidized serum albumin

## Abstract

The identification of natural bioactive compounds, able to counteract the abnormal increase of oxidative stress and inflammatory status in chronic degenerative non-communicable diseases is useful for the clinical management of these conditions. We tested an oral food supplement (OFS), chemically characterized and evaluated for in vitro and in vivo activity. Vitamin C, analyzed by High Performance Liquid Chromatography-Diode Array Detector (HPLC-DAD), was 0.19 mg/g in rosehip dry extract and 15.74 mg/capsule in the OFS. The identification of polyphenols was performed by HPLC-DAD; the total antioxidant capacity was assessed by Folin–Ciocalteu test. Total polyphenols were 14.73 mg/g gallic acid equivalents (GAE) for rosehip extract and 1.93 mg/g GAE for OFS. A total of 21 chronic kidney disease (CKD) patients and 10 healthy volunteers were recruited. The evaluation of routine laboratory and inflammatory parameters, erythrocyte glutathione transferase (e-GST), human oxidized serum albumin (HSAox), and assessment of body composition were performed at two different times, at baseline and after 5 weeks of OFS assumption. In the study, we highlighted a significant decrease of traditional inflammatory biomarkers (such as C-reactive protein, erythrocyte sedimentation rate, platelet to lymphocyte ratio) and other laboratory parameters like e-GST, azotaemia, and albuminuria after OFS treatment in CKD patients. Moreover, we demonstrated a lipid profile improvement in CKD patients after OFS supplementation.

## 1. Introduction

Chronic kidney disease (CKD) is a major health problem with a considerable worldwide impact and the onset is correlated to the spread of its risk factors such as obesity, metabolic syndrome, arterial hypertension, and diabetes mellitus (DM) [1,2].

CKD is directly related with cardiovascular (CV) mortality and morbidity. The latter displayed a 10 to 20-fold increase respect to general population [3,4]. This phenomenon cannot only be explained with CV traditional risk factors, but it needs a more accurate assessment that takes into consideration the uremic CV risk factors. Uremic CV risk factors that exert a key role are chronic low-grade inflammatory status [5], alteration of calcium-phosphorus metabolism [6,7,8,9], hyperhomocysteinemia [10], malnutrition [11], uremic sarcopenia [12,13,14], and oxidative stress (OS) [15,16].

The increase in OS is caused by an imbalance between antioxidant defenses and free radical production. Oxygen free radicals are formed in the mitochondria related to aging, DM, CKD, and inflammation [17]. The increased concentration of pro-oxidant substances influences cellular communication; in the kidney it promotes apoptosis and cell senescence, determining reduced regenerative cellular capacity and fibrosis [18,19].

Vitamin C is one of the possible factors linked to the presence of OS in uremic patients [20,21]. In fact, it is common to observe a vitamin C deficiency in this population caused by restricted intake of vitamin C-rich foods and by its increased metabolism induced by the chronic low-grade inflammatory status [22]. Nowadays, several studies are in progress to formulate oral food supplements (OFS) with high ascorbic acid content from natural citrus fruit and rosehip extracts [23,24] rather than synthetic ascorbic acid. In this context, it would be very useful to identify natural bioactive compounds, which are able to counteract the abnormal increase of OS and inflammatory status in chronic degenerative non-communicable diseases [25,26].

In this study an OFS, enriched in standardized and characterized rosehip natural extract, was tested in both CKD patients and healthy subjects. In particular, the standardized rosehip extract, was chemically characterized for its polyphenols and vitamin C contents.

The aim of this study is to evaluate the effects of Siuper^®^, an OFS (based on *Echinacea angustifolia*, zinc, rosehip, propolis, and royal jelly) at the dosage of 400 mg per three die, on the inflammatory status, OS, blood toxicity, body composition, and renal function both in CKD patients and in the healthy subjects. For evaluation of inflammatory status we examined the C-reactive protein (CRP), the erythrocyte sedimentation rate (ESR), platelet-to-lymphocyte ratio (PLR), neutrophil-to-lymphocyte ratio (NLR), and lymphocyte-to-monocyte ratio (LMR) [27].

Furthermore, two biomarkers of blood toxicity and OS such as the erythrocyte glutathione transferase (e-GST) and human oxidized serum albumin (HSAox), respectively, were also monitored in the present study [19,28]. e-GST was selected due to its remarkable sensitivity in detecting large spectrum of blood toxins [12] in a temporal span of a few weeks. Conversely, HSAox is a short-term biomarker of OS and its blood concentration may change in the temporal span of a few hours [15].

## 2. Results

### 2.1. Chemical Characterization of the Rosehip Dry Extract and Food Supplement

The results are calculated with external calibration curves, usually in gallic acid, and expressed as mg/g gallic acid equivalents (GAE). The in vitro antioxidant activity shows a correlation with total phenols and minor polar compounds as confirmed by previous studies carried out by comparing different electron transfer reaction assays (e.g., FRAP, TEAC, and ORAC) and in vitro assays on human low density lipoproteins (LDL) [29,30,31]. The total phenols and polyphenols contents are 14.73 mg/g GAE for the rosehip extract and 1.93 mg/g GAE for Siuper^®^ food supplement. The HPLC-DAD profile allowed the identification of the main polyphenolic subclasses in the rosehip dry extract and in the Siuper^®^. Procyanidins are the main polyphenols present, but also hydroxycynnamic derivatives and flavonols (mainly quercetin and kaempferol derivatives) were identified, such as tiliroside, a glycosyloxyflavone deriving from a kaempferol unit and a *p*-coumaric acid. The total vitamin C content by HPLC-DAD analysis was 0.19 mg/g of powder in the rosehip dry extract, and 39.34 mg/g of powder in the Siuper^®^ (15.74 mg/capsule).

### 2.2. Effect of Oral Food Supplement in CKD Patients and Healthy Subjects

The epidemiological features and statistical analysis to evaluate the homogeneity of the study groups were reported in Table 1.

In Table 2, we summarized and compared routine laboratory parameters of CKD patients at different times of the study (T0 and T1). We observed a significant reduction in albuminuria (10 (0–100) mg/dL vs. 0 (0–70) mg/dL, *p* = 0.0234) and azotaemia (79 ± 29 mg/dL vs. 73 ± 25 mg/dL, *p* = 0.0024) after OFS treatment. We highlighted a reduction of triglycerides and transferrin (137 ± 57 mg/dL vs. 115 ± 53 mg/dL, *p* = 0.0041; 239 ± 42 mg/dL vs. 227 ± 33 mg/dL, *p* = 0.0428, respectively) at the end of the study.

Moreover, we did not observe a significant impact of OFS treatment on phosphate, calcium, and parathyroid hormone (PTH) levels. In the healthy subjects we did not observe statistically significant differences in routine laboratory parameters, as reported in Table 3.

In Table 4, we illustrated the body composition parameters of CKD group before and after OFS treatment; all the parameters examined did not undergo statistically significant changes.

Blood pressure did not show changes after treatment, in both CKD patients and in the healthy subjects (Table 5 and Table 6).

Interestingly, in CKD patients a significant reduction in inflammatory biomarkers examined was observed; in fact, CRP and ESR decreased significantly at T1 (5 weeks of OFS after treatment) as reported in Table 7.

Moreover, a significant reduction of e-GST activity was observed at T1 (Table 7).

**Figure 1:**
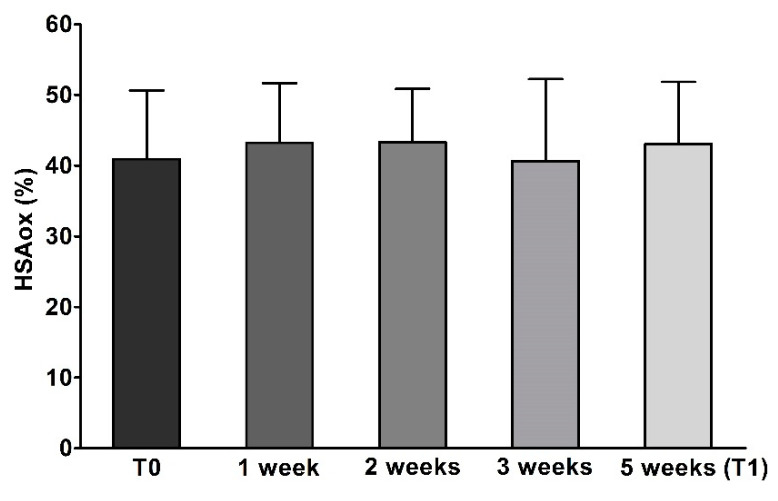
Human oxidized serum albumin in chronic kidney disease patients. The bars (five shades of grey) represent the values HSAox determined at various times (T0, 1 week, 2 weeks, 3 weeks, and 5 weeks—T1) for CKD patients (*N* = 21). Values are reported as mean ± standard deviation.

Finally, HSAox did not show any significant variation during four distinct assays from weeks 1 to 5 of OFS treatment (Figure 1 and Table 7).

The inflammatory and oxidative stress biomarkers of the healthy subjects are reported in the Table 8. We did not observe any statistically significant difference after OFS treatment.

In healthy subjects, significant changes were only observed in body composition. In particular, an increased phase angle (°), FFM (%), MM (%), and TBW (%) were shown after OFS treatment (Table 9). Notably, a significant reduction in FM (%) was observed in healthy subjects. Finally, in healthy subjects, no statistically significant change was observed in the examined laboratory parameters (Table 9).

We also evaluated the impact of OFS on immune system and inflammatory status (Table 10 and Table 11). We observed in CKD patients a significant reduction of PLR (146.2 (86.2–398.7) vs. 122.0 (54.7–337.5), *p* = 0.004) (Table 10). In the healthy subjects, we did not observe statistically significant differences in other biological markers examined (Table 11).

**Figure 2:**
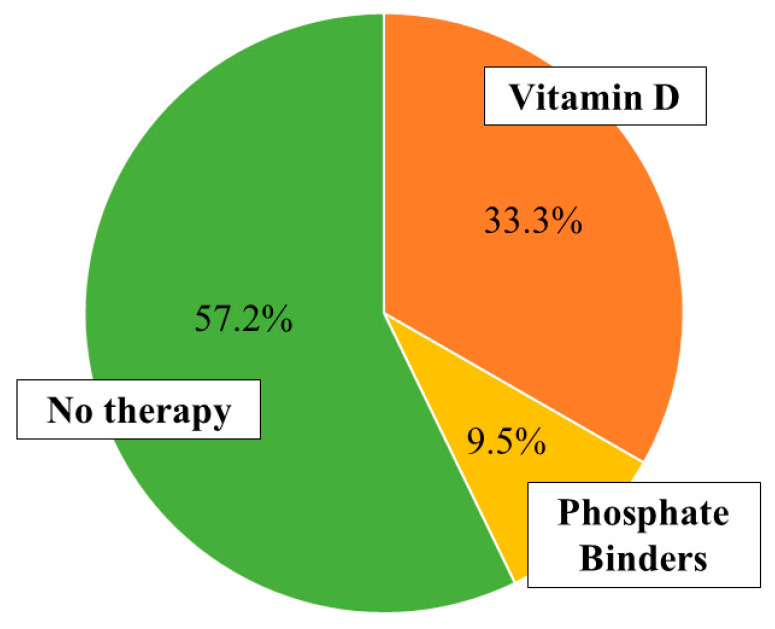
Pharmacological therapy on calcium-phosphorus metabolism.

As in a previous paper an inverse correlation between vitamin D concentration and inflammatory state has been described (assessed by PLR and NLR) [32], we considered it appropriate to include a serum concentration of vitamin D < 30 ng/mL among the enrolment exclusion criteria. In addition, we collected pharmacological anamnesis regarding therapeutic approach for the management of calcium-phosphorus metabolism (Figure 2).

In order to confirm that the observed improvements in the health status of studied populations were due to the intake of OFS and not to lifestyle changes (such as eating habits and physical activity), we administered to all enrolled subjects (at T0 and T1) the PREDIMED and IPAQ questionnaires. These questionnaires did not show, between the two observation times, statistically significant changes, confirming the putative health effects induced by OFS treatment (Table 12 and Table 13).

## 3. Discussion

CKD patients are characterized by an on-going chronic inflammatory state, enhanced OS, and impaired immune response [33]. These factors are interconnected with an increased CV risk. The identification of a natural OFS, not synthetically produced, standardized in polyphenol and vitamin C contents, which could counteract both inflammation, OS, and modulate the immune response, should be useful. OFS would be a valid tool to improve the quality of life and reduce the CV comorbidity in nephropathic patients on conservative therapy. In our study, we highlighted that the assumption of OFS Siuper^®^ for 5 weeks significantly decreases traditional inflammatory biomarkers such as CRP, ESR, and a new biomarker of inflammatory status such as PLR. The latter has been correlated to the proinflammatory and prothrombotic status [34]. Moreover, previous studies have demonstrated that enhanced CRP levels, the acute phase response protein, are linked to higher hospitalization rate and lower serum albumin levels in pre-dialysis patients [35]. High CRP values are related to an increased carotid-intima media area in pre-dialysis patients, highlighting a direct correlation between the concentration of CRP and oxidized LDL [36]. Therefore, a possible relationship between the increase in OS and inflammation, in uremic patients, appears evident. However, in this study a significant reduction in terms of OS, monitored by HSAox, was not observed. This result could be interpreted with a limited time of administration of the OFS or with the need to increase the content of polyphenols and vitamin C for each OFS dose.

The OFS crucial ingredients with potential therapeutic impact are rosehip, zinc, and *Echinacea angustifolia.* In particular, previous studies showed that zinc levels inversely correlate with pro-inflammatory cytokines concentration, such as IL-6, IL-8, and tumor necrosis factor-α (TNF-α) [37,38], regulating their gene expression [39]. In our OFS the dose of zinc for each capsule is 4.5 mg; therefore, the daily zinc dose taken was 13.5 mg, following with the maximum tolerable daily intake (25 mg/day), as suggested by Italian LARN [40].

Therefore, the reduction of the inflammatory biomarkers, observed after 5 weeks of OFS treatment, can be partly due to the zinc anti-inflammatory action. Moreover, also *Echinacea angustifolia* showed anti-inflammatory properties as reported in an animal study by Aarland et al. [41]. Thus confirming previous data showing a reduction in inflammatory cell infiltration, in an in vitro study [42].

Finally, rosehip, rich in vitamin C and polyphenolic compounds, has an antioxidant and anti-inflammatory effect [43]. An interesting study has demonstrated its anti-inflammatory action on polymorphonuclear chemotaxis, highlighting that this effect is in a “dose-dependent” manner [44]. An additional effect showed by an aqueous extract from rosehip was the inhibition, in vitro, of COX-1 and COX-2 [45].

In this study, a significant reduction of e-GST activity, after 5 weeks of OFS supplementation, was observed in CKD patients. The activity of this enzyme can be related to the presence of uremic toxins, as previously demonstrated in uremic patients [46,47]. In our study, the reduction of e-GST after OFS treatment in CKD patients (Table 7) and the lack of HSAox variation (Figure 1 and Table 7) indicate that Siuper^®^ mainly has a detoxifying action. In fact, additional parameters confirm the benefit of OFS in CKD patients through the significant reduction of azotaemia and albuminuria at T1. Decreased azotaemia can be viewed as an expression of an improvement in the state of blood toxicity, confirming the possible detoxifying action of the OFS; whilst albuminuria is a useful indicator of the progression of CKD and is directly correlated with CV risk [48].

This detoxifying action of OFS would seem to be caused by its content in polyphenols and vitamin C, as both are able to reduce the formation of reactive oxygen species (ROS), playing a key role in the cell’s detoxification [30,49]. Moreover, a study in healthy young adults showed that a low serum concentration of ascorbic acid is correlated with the concentration of glutathione, thiols, and total antioxidant capacity [50].

We also observed an improvement in the lipid profile with a statistically significant reduction in blood triglyceride values at the end of the OFS treatment (Table 2). This result confirms the possible beneficial activity of the OFS in reducing CV risk [51]. High blood levels of triglycerides predispose to a higher risk of CV events, especially if associated with other risk factors such as high LDL cholesterol, obesity, and DM [52,53].

This OFS effect could be induced by rosehip as evidenced by preceding studies. In fact, in mice the administration of extracts from rosehip at dose 100/200 mg/kg/day caused, after 14 days, a reduction of plasma triglyceride and free fatty acid levels. This action would seem to be exercised by its polyphenolic fraction, in particular *trans*-tiliroside, which would seem to promote lipid metabolism [54].

Another study confirms the lipid-lowering action of rosehip, in fact in the rats the concentration of cholesterol and triglyceride were significantly lower after a rosehip oil diet compared to the healthy subjects [55].

A statistically significant reduction in transferrin and an increase in ferritin were also shown, while remaining within the normal range (Table 2). Their synthesis is self-regulated in relation to the martial state, but their concentration can be influenced by other factors unrelated to the iron levels, such as phlogistic status, hepatopathies [56], malnutrition, and nephrotic syndrome [57].

With regards to the possible effects of OFS on body composition in CKD patients, no statistically significant changes were observed. In contrast, in healthy subjects we observed an improvement in body composition. Specifically, a reduction in FM and an increase in phase angle, TBW, FFM, MM, and BCMI (Table 9). Therefore, we can hypothesize that OFS can act at the metabolic level by stimulating energy metabolism [12]. In CKD patients, this improvement is not observed (Table 4) supposedly since this condition induces, especially in the final stages, a state of metabolic acidosis, which is responsible for stimulating the ubiquitin-proteasome pathway, which can induce muscle proteolysis [58,59]. Therefore, in order to assess the possible beneficial effect of OFS on body composition, it will be necessary to conduct a randomized clinical trial in which the metabolic acidosis will be also evaluated. Moreover, the body composition will be determined not only through bioelectrical impedance analysis (BIA) but also through further diagnostic methods, such as dual-energy X-ray absorptiometry.

In order to demonstrate the correlation between OS decrease and the administration of natural bioactive compounds (such as polyphenols and vitamin C) in CKD, further studies are in progress including the evaluation of plasma OS, total free radicals and total plasma antioxidant capacity in addition to the parameters described in this study.

In conclusion, this OFS would seem useful in the treatment of chronic inflammatory status and blood toxicity in CKD patients. The promising results obtained in our pilot study laid the foundation for a further randomized clinical trial, conducted on a larger number of patients that will be needed to confirm these results.

## 4. Materials and Methods

### 4.1. Oral Food Supplement, Analysis of Vitamin C, Polyphenol Total Content and Antioxidant Capacity In Vitro

The OFS was the Siuper^®^ Marispharma, Frosinone, Italy, based on *Echinacea angustifolia,* zinc, rosehip, propolis, and royal jelly. OFS was administered at the dose of 400 mg per three die, for the evaluation of its possible effects on the inflammatory status, OS, blood toxicity, body composition, and renal function in CKD patients and in the healthy subjects, in our pilot study. The analyzed rosehip dry extract used as an ingredient was supplied by the company Marispharma.

For the extraction of vitamin C, 400 mg of OFS powder or rosehip dry extract were stirred in 4.0 mL 70:30 EtOH:H_2_O (pH 2.4 by addition of HCOOH) at room temperature for 30 min, centrifuged at 14,000 rpm for 5 min and analyzed. For the extraction of polyphenols, 500 mg of OFS powder or rosehip dry extract were stirred in 25.0 mL of 70:30 EtOH:H_2_O (pH 3.2 by addition of HCOOH) for 24 h, centrifuged at 14,000 rpm for 5 min and analyzed.

The HPLC-DAD analyses were performed with a liquid chromatography HP-1100 equipped with a DAD detector (Agilent-Technologies, Santa Clara, CA, USA) and a Zorbax SB-aq C18 (150 × 4.6 mm i.d. 5 μm) (Agilent-Technologies, Santa Clara, CA, USA) column. For ascorbic acid a 0.4 mL/min flow was applied for 8 min with a mobile phase 95% H_2_O (pH 3.2 by addition of HCOOH) (A) 5% CH_3_CN (B). For the analysis of polyphenols, a multistep linear gradient was applied from 100% A to 100% B in 43 min with a 0.4 mL/min flow.

The qualitative analysis was performed according to chromatographic and spectrophotometric data, by comparison with the specific standards available. The quantification of vitamin C was performed by using a 5-points calibration curve (r^2^ ≥ 0.999) in ascorbic acid at 260 nm.

Total phenols and polyphenols were evaluated by spectrophotometric Folin-Ciocalteu assay, measuring absorbance at 725 nm of a sample solution containing Folin-Ciocalteu reagent, 20% Na_2_CO_3_ after 40 min incubation. The calibration curve was obtained using five gallic acid solutions at different concentrations. The phenols content of each sample is expressed as GAEs and correlated with the in vitro antioxidant activity [30,31].

All analyses were carried out in triplicate; the results are given as means and the standard error was <3%.

### 4.2. Chemicals and Reagents

Cystamine, 1-chloro-2,4-dinitrobenzene (CDNB), ethylenediaminetetraacetic acid (EDTA), 5,5′-dithiobis(2-nitrobenzoic acid) (DTNB) (Ellman’s reagent), glutathione (GSH), formic acid (HCOOH) HPLC grade, acetonitrile (CH_3_CN) HPLC grade, ethanol (EtOH) HPLC grade, ascorbic acid, Folin–Ciocalteu reactive, sodium carbonate (Na_2_CO_3_), and all other reagents were purchased from Sigma-Aldrich (St. Louis, MO, USA).

### 4.3. Patients

Men and women aged 18–80 years were considered suitable for the study. The study protocol complied with the declaration of Helsinki and was appointed by the Ethical Committee of Fondazione Policlinico Tor Vergata (PTV) of Rome.

A written fully informed consent was provided to all CKD patients and healthy subjects before enrolment into the study. Exclusion criteria were cancer, virus hepatitis B and C, rheumatologic disorders (e.g., systemic lupus erythematosus), pregnancy, chronic maintenance hemodialysis, body mass index (BMI) <18.5 kg/m^2^, vitamin D concentration <30 ng/mL, and oral supplements in the last 3 months.

A total of 21 patients (mean age 68.7 ± 10 years), affected by CKD (stage I–IV according to the National Kidney Foundation Kidney—Disease Outcomes Quality Initiative guidelines [60]), were recruited from Centre of Hypertension and Nephrology Unit of Fondazione PTV, Rome.

In CKD patients, the primary causes of renal failure were glomerulonephritis (10%), nephroangiosclerosis (48%), diabetic nephropathy (14%), chronic pyelonephritis (5%), autosomal dominant polycystic kidney disease (9%), and other causes (14%).

Participation in the study included a complete medical history to gather information about health status, current medications, eating habits, alcohol drinking, smoking, and family history for chronic diseases.

Ten healthy volunteers matched for age, sex, and body composition parameters such as weight, height, and BMI, constituted the healthy subjects.

All enrolled subjects (CKD patients and healthy subjects) were treated for 5 weeks with OFS (400 mg per three die).

A complete evaluation of laboratory parameters and body composition were conducted at two different times of the OFS treatment, at baseline (T0) and after 5 weeks (T1). Only HSAox was monitored every week.

### 4.4. Anthropometric Measurements and Body Composition Assessment

Anthropometric parameters of all the participants were recorded according to standard methods [61]. Body weight (kg) was measured to the nearest 0.01 kg, using a balance scale (Seca 711, Hamburg, Germany). Height (m) was measured using a stadiometer to the nearest 0.1 cm (Seca 220, Hamburg, Germany). BMI was calculated as body weight divided by height squared (kg/m^2^).

For evaluation of body composition, all enrolled subjects performed BIA. Resistance (R), reactance (Xc), and phase angle at 50 KHz frequency were measured using a BIA 101S instruments (Akern/RIL System-Florence) [62].

### 4.5. Laboratory Parameters

For measurement of laboratory parameters, we used an automated hematology analyzer XE-2100 (Sysmex, Kobe, Japan) for the determination of hemoglobin (Hb). All routine parameters were determined using Dimension VISTA 1500 (Siemens Healthcare Diagnostics, Milano, Italy).

The lipid profile, like total-cholesterol (TC), triglyceride, low-density lipoprotein cholesterol (LDL), and high-density lipoprotein cholesterol (HDL), was determined by standard enzymatic colorimetric techniques (Roche modular P800, Roche diagnostics, Indianapolis, IN, USA).

### 4.6. Questionnaires

Two questionnaires were administered to the enrolled subjects: Prevención con Dieta Mediterránea (PREDIMED) [63] and International Physical Activity Questionnaire (IPAQ) [64] at the beginning and at the end of the study.

The first was administered to assess the adherence to Mediterranean diet, the second was administered to evaluate the degree of physical activity before and after OFS treatment. Both questionnaires were administered in order to avoid possible biases related to eating habits or physical activity on examined parameters in the study.

### 4.7. Erythrocyte Glutathione Transferase Activity

e-GST activity was assessed using a spectrophotometric assay at 340 nm (37 °C), with an Uvikon 941 Plus spectrophotometer (Kontron Instruments, Watford, Herts, UK). Shortly, 40 μL of whole blood was diluted into 1 mL of bi-distilled water causing immediate erythrocyte hemolysis. After that, 100 µL of hemolyzed blood was diluted to a final volume of 1 mL containing 1 mM GSH, 1 mM CDNB in 0.1 M potassium phosphate buffer pH 6.5 [65]. Data was reported as enzyme units (U) per gram of hemoglobin (Hb) (U/gHb): one unit is the amount of enzyme that catalyzes the conjugation of 1 micromole of GSH to CDNB in 1 min at 37 °C [66].

### 4.8. Human Oxidized Serum Albumin

HSAox was determined by subtracting reduced HSA values from the total HSA. Reduced HSA was determined by exploiting the fast reaction of cystamine with Cys34, the only free cysteine in albumin that is partially present as mixed disulfide (generally with cysteine) depending on the redox status of the human serum. The released cysteamine is stoichiometric with Cys34 and it can be determined with DTNB (ε_412nm_ = 14,100 M^−1^ cm^−1^). The assay was performed with a Kontron Uvikon 941 Plus spectrophotometer (Kontron Instruments) at 412 nm at 25 °C. A human serum volume of 50 μL was diluted in 890 μL of potassium phosphate buffer 0.1 M pH 8.0, recording an autozero sample. Then, 50 μL of DTNB (50 μM final concentration) and 10 μL of cystamine (1 mM final concentration) were added to the solution. After an incubation of 15 min at room temperature, the absorbance was recorded [46]. Total HSA was determined according to the standard laboratory protocols.

### 4.9. Statistical and Graphical Analysis

Data is reported as mean ± standard deviation for parametric variables and as median (range minimum–maximum) for non-parametric variables. All continuous variables were checked for normality using Kolmogorov–Smirnov test. Differences between the baseline and the final outcomes for parametric values were tested with a paired *t*-test. The non-parametric variables data was analyzed for significance with the Wilcoxon test. The minimal level of significance of the differences was fixed at *p* < 0.05. Comparison among groups was performed with the univariate ANOVA with a covariate for continuous parametric variables. In our study, every enrolled subject was the control of himself. Furthermore, the short matrices of data of PREDIMED and IPAQ were analyzed with McNemar’s test [67]. This analysis was performed using the Statistical Package for the Social Sciences Windows, version 15.0 (SPSS, Chicago, Illinois, USA). The graphic and results visualization were obtained by GraphPad Prism (La Jolla, CA, USA).

## Figures and Tables

**Table 1:** Epidemiological findings of study population and evaluation of the homogeneity of the study groups.

	CKD Patients	Healthy Subjects	*p* (ANOVA Test)
***N***	21	10	
**Gender (male/female)**	13/8	6/4	ns
**Age (years)**	68.7 ± 10.0 ^a^	67.1 ± 4.6 ^a^	ns
**Height (m)**	1.64 ± 0.10 ^a^	1.67 ± 0.1 ^a^	ns
**Weight (kg)**	73.6 ± 12.9 ^a^	73.9 ± 5.5 ^a^	ns
**BMI (kg/m^2^)**	27.3 ± 4.8 ^a^	26.7 ± 3.0 ^a^	ns

^a^ Data expressed as mean ± standard deviation; Abbreviations: BMI, body mass index; CKD, Chronic kidney disease; ns, not significant.

**Table 2:** Routine laboratory parameters of chronic kidney disease (CKD) patients.

Laboratory Parameter	T0	T1	T0 vs. T1
**Creatinine (mg/dL)**	2.0 (1.1–4.5) ^a^	1.9 (1.1–4.7) ^a^	ns ^b^
**e-GFR (mL/min/1.73 m^2^)**	33 ± 16 ^c^	33 ± 16 ^c^	ns ^d^
**Albuminuria (mg/dL)**	10 (0–100) ^a^	0 (0–70) ^a^	*p* = 0.0234 ^b^
**Azotaemia (mg/dL)**	79 ± 29 ^c^	73 ± 25 ^c^	*p* = 0.0024 ^d^
**Albumin (g/dL)**	4.3 ± 0.2 ^c^	4.2 ± 0.1 ^c^	ns ^d^
**Hemoglobin (g/dL)**	12.9 ± 2.1 ^c^	12.7 ± 2.3 ^c^	ns ^d^
**Sodium (mEq/L)**	140 (133–144) ^a^	140 (134–143) ^a^	ns ^b^
**Potassium (mEq/L)**	4.8 ± 0.5 ^c^	4.8 ± 0.4 ^c^	ns ^d^
**Calcium (mg/dL)**	9.7 (7.9–10.5) ^a^	9.7 (8.3–10.5) ^a^	ns ^b^
**Phosphorus (mg/dL)**	3.4 ± 0.6 ^c^	3.4 ± 0.6 ^c^	ns ^d^
**PTH (pg/mL)**	141.29 ± 76.3 ^c^	137.3 ± 81.3 ^c^	ns ^d^
**TC (mg/dL)**	174 ± 36 ^c^	167 ± 33 ^c^	ns ^d^
**HDL (mg/dL)**	51 ± 13 ^c^	49 ± 12 ^c^	ns ^d^
**Triglycerides (mg/dL)**	137 ± 57 ^c^	115 ± 53 ^c^	*p* = 0.0041 ^d^
**LDL (mg/dL)**	103 ± 45 ^c^	97 ± 33 ^c^	ns ^d^
**Sideremia (μg/dL)**	70 ± 24 ^c^	70 ± 31 ^c^	ns ^d^
**Ferritin (ng/mL)**	85 (7–561) ^a^	95 (7–541) ^a^	*p* = 0.0289 ^b^
**Transferrin (mg/dL)**	239 ± 42 ^c^	227 ± 33 ^c^	*p* = 0.0428 ^d^
**Uric acid (mg/dL)**	6.7 (2.4–9.0) ^a^	6.7 (1.8–9.6) ^a^	ns ^b^

^a^ Data expressed as median and the minimum-maximum range is shown in brackets; ^b^ Applied test: Wilcoxon test; ^c^ Data expressed as mean ± standard deviation; ^d^ Applied test: *t*-test for paired data. Values of *p* ≤ 0.05 are considered statistically significant. Abbreviations: e-GFR, estimated glomerular filtration rate; TC, total cholesterol; HDL, high-density lipoprotein; LDL, low-density lipoprotein; ns, not significant; PTH, parathyroid hormone.

**Table 3:** Routine laboratory parameters of healthy subjects.

Laboratory Parameter	T0	T1	T0 vs. T1
**Creatinine (mg/dL)**	0.79 ± 0.14 ^a^	0.8 ± 0.14 ^a^	ns ^b^
**e-GFR (mL/min/1.73 m^2^)**	93.5 ± 20.4 ^a^	92.1 ± 14.3 ^a^	ns ^b^
**Albuminuria (mg/dL)**	2.1 ± 4.4 ^a^	0.0 ± 0.0 ^a^	ns ^b^
**Azotaemia (mg/dL)**	32.6 ± 8.0 ^a^	34.8 ± 4.7 ^a^	ns ^b^
**Albumin (g/dL)**	4.3 ± 0.2 ^a^	4.4 ± 0.3 ^a^	ns ^b^
**Hemoglobin (g/dL)**	14.3 ± 1.5 ^a^	14.3 ± 1.6 ^a^	ns ^b^
**Sodium (mEq/L)**	141.7 ± 2.6 ^a^	140 ± 2.9 ^a^	ns ^b^
**Potassium (mEq/L)**	4.5 ± 0.4 ^a^	4.5 ± 0.3 ^a^	ns ^b^
**Calcium (mg/dL)**	9.3 ± 0.4 ^a^	9.7 ± 0.4 ^a^	ns ^b^
**Phosphorus (mg/dL)**	3.5 ± 0.5 ^a^	3.6 ± 0.5 ^a^	ns ^b^
**PTH (pg/mL)**	56.2 ± 10.3 ^a^	59.5 ± 7.1 ^a^	ns ^b^
**TC (mg/dL)**	196.3 ± 26.5 ^a^	188.9 ± 19.8 ^a^	ns ^b^
**HDL (mg/dL)**	50.3 ± 12.5 ^a^	52.9 ± 11.9 ^a^	ns ^b^
Triglycerides (mg/dL)	99.5 ± 32.6 ^a^	92.4 ± 34.0 ^a^	ns ^b^
**LDL (mg/dL)**	116.4 ± 21.4 ^a^	120.7 ± 18.2 ^a^	ns ^b^
**Sideremia (μg/dL)**	89.5±25.1 ^a^	85.7 ± 20.7 ^a^	ns ^b^
**Ferritin (ng/mL)**	195.2 ± 163.9 ^a^	179.4 ± 139.7 ^a^	ns ^b^
**Transferrin (mg/dL)**	255.7 ± 39.6 ^a^	248.5 ± 34.7 ^a^	ns ^b^
**Uric acid (mg/dL)**	5.0 ± 1.0 ^a^	4.9 ± 0.9 ^a^	ns ^b^

^a^ Data expressed as mean ± standard deviation; ^b^ Applied test: *t*-test for paired data. Values of *p* ≤ 0.05 are considered statistically significant. Abbreviations: e-GFR, estimated glomerular filtration rate; TC, total cholesterol; HDL, high-density lipoprotein; LDL, low-density lipoprotein; ns, not significant; PTH, parathyroid hormone.

**Table 4:** Body composition parameters of CKD patients.

Body Composition Parameter	T0	T1	T0 vs. T1
**Resistance (ohm)**	503 ± 95 ^a^	503 ± 85 ^a^	ns ^b^
**Reactance (ohm) **	37 ± 6 ^a^	38 ± 8 ^a^	ns ^b^
**Phase angle (°)**	4.2 ± 0.8 ^a^	4.4 ± 1.0 ^a^	ns ^b^
**BMI (kg/m^2^)**	27.2 ± 5.1 ^a^	27.1 ± 5.2 ^a^	ns ^b^
**Weight (kg)**	73.2 ± 13.3 ^a^	73.3 ± 12.7 ^a^	ns ^b^
**TBW (%)**	55.7 ± 7.3 ^a^	55.5 ± 6.8 ^a^	ns ^b^
**ICW (%)**	43.6 ± 5.7 ^a^	44.1 ± 7.1 ^a^	ns ^b^
**ECW (%)**	54.5 ± 10.1 ^a^	55.5 ± 6.9 ^a^	ns ^b^
**FM (%)**	30.2 ± 9.4 ^a^	30.5 ± 8.9 ^a^	ns ^b^
**FFM (%)**	70.2 (46.6–88.1) ^c^	69.8 (48.1–86.7) ^c^	ns ^d^
**MM (%)**	38.6 ± 8.1 ^a^	38.9 ± 7.8 ^a^	ns ^b^
**BCMI (kg/m^2^)**	7.9 ± 1.9 ^a^	8.1 ± 2.0 ^a^	ns ^b^

^a^ Data expressed as mean ± standard deviation; ^b^ Applied test: *t*-test for paired data; ^c^ Data expressed as a median and the minimum–maximum range is shown in brackets; ^d^ Applied test: Wilcoxon test. Values of *p* ≤ 0.05 are considered statistically significant; Abbreviations: BMI, body mass index; TBW, total body water; ICW, intra cell water; ECW, extra cell water; FM, fat mass; FFM, fat free mass; MM, muscle mass; BCMI, body cellular mass index; ns, not significant.

**Table 5:** Blood pressure parameters of CKD patients.

Blood Pressure Parameter	T0	T1	T0 vs. T1
**Systolic Pressure (mmHg)**	135 ± 17 ^a^	135 ± 14 ^a^	ns ^b^
**Diastolic Pressure (mmHg)**	77 ± 12 ^a^	75 ± 10 ^a^	ns ^b^

^a^ Data expressed as mean ± standard deviation; ^b^ Applied test: *t*-test for paired data. Values of *p* ≤ 0.05 are considered statistically significant; Abbreviations: ns, not significant.

**Table 6:** Blood pressure parameters of healthy subjects.

Blood Pressure Parameter	T0	T1	T0 vs. T1
**Systolic Pressure (mmHg)**	117.5 ± 11.3 ^a^	113.6 ± 9.8 ^a^	ns ^b^
**Diastolic Pressure (mmHg)**	73.8 ± 6.6 ^a^	75.1 ± 12.0 ^a^	ns ^b^

^a^ Data expressed as mean ± standard deviation; ^b^ Applied test: *t*-test for paired data; Values of *p* ≤ 0.05 are considered statistically significant; Abbreviations: ns, not significant.

**Table 7:** Inflammatory and oxidative stress biomarkers of CKD patients.

Biomarker	T0	T1	T0 vs. T1
**CRP (mg/L)**	2.6 (0.2–48.1) ^a^	2.3 (0.4–34.6) ^a^	*p* = 0.0121 ^b^
**ESR (mm/h)**	40 ± 27 ^c^	34 ± 19 ^c^	*p* = 0.0446 ^d^
**e-GST (U/g Hb)**	10.0 (6.1–23.5) ^a^	9.1 (4.8–25.8) ^a^	*p* = 0.0296 ^b^
**HSAox (%)**	41 ± 10 ^c^	43 ± 8 ^c^	ns ^d^

^a^ Data expressed as a median and the minimum–maximum range is shown in brackets; ^b^ Applied test: Wilcoxon test; ^c^ Data expressed as mean ± standard deviation; ^d^ Applied test: *t*-test for paired data. Values of *p* ≤ 0.05 are considered statistically significant; Abbreviations: CRP, C-reactive protein; ESR, erythrocyte sedimentation rate; e-GST, erythrocyte glutathione transferase; HSAox, human oxidized serum albumin; ns, not significant.

**Table 8:** Inflammatory and oxidative stress biomarkers of healthy subjects.

Biomarker	T0	T1	T0 vs. T1
**CRP (mg/L)**	2.8 ± 2.9 ^a^	1.8 ± 1.8 ^a^	ns ^b^
**ESR (mm/h)**	8.5 ± 2.1 ^a^	5.0 ± 4.2 ^a^	ns ^b^
**e-GST (U/g Hb)**	7.6 ± 2.6 ^a^	7.3 ± 2.7 ^a^	ns ^b^
**HSAox (%)**	30.7 ± 5.5 ^a^	29.7 ± 3.2 ^a^	ns ^b^

^a^ Data expressed as mean ± standard deviation; ^b^ Applied test: *t*-test for paired data; Values of *p* ≤ 0.05 are considered statistically significant; Abbreviations: CRP, C-reactive protein; ESR, erythrocyte sedimentation rate; e-GST, erythrocyte glutathione transferase; HSAox, human oxidized serum albumin; ns, not significant.

**Table 9:** Body composition parameters of healthy subjects.

Body Composition Parameter	T0	T1	T0 vs. T1
**Resistance (ohm)**	546 ± 44 ^a^	543 ± 45 ^a^	ns ^b^
**Reactance (ohm) **	53 ± 12 ^a^	53 ± 11 ^a^	ns ^b^
**Phase angle (°)**	5.0 (4.5–7) ^c^	5.2 (4.7–6.9) ^c^	*p* = 0.0195 ^d^
**BMI (kg/m^2^)**	27.4 (22.5–27.6) ^c^	27.0 (22.4–27.8) ^c^	ns ^d^
**Weight (kg)**	73.8 (70.1–92.5) ^c^	73.5 (69.0–92.3) ^c^	ns ^d^
**TBW (%)**	52.4 ± 4.1 ^a^	53.1 ± 3.9 ^a^	*p* = 0.0029 ^d^
ICW (%)	49.3 (46.5–58.5) ^c^	50.3 (46.9–58.0) ^c^	ns ^d^
**ECW (%)**	50.7 (41.5–53.5) ^c^	49.7 (42.0–54.1) ^c^	ns ^d^
**FM (%)**	28.4 ± 5.7 ^a^	27.7 ± 5.2 ^a^	*p* = 0.0049 ^b^
**FFM (%)**	71.5 ± 5.7 ^a^	72.4 ± 5.1 ^a^	*p* = 0.0055 ^b^
**MM (%)**	45.0 ± 4.7 ^a^	45.7 ± 4.0 ^a^	*p* = 0.0226 ^b^
**BCMI (kg/m^2^)**	9.2 ± 0.9 ^a^	9.4 ± 0.8 ^a^	*p* = 0.0163 ^b^

^a^ Data expressed as mean ± standard deviation; ^b^ Applied test: *t*-test for paired data; ^c^ Data expressed as a median and the minimum–maximum range is shown in brackets; ^d^ Applied test: Wilcoxon test; Values of *p* ≤ 0.05 are considered statistically significant; Abbreviations: BMI, body mass index; TBW, total body water; ICW, intra cell water; ECW, extra cell water; FM, fat mass; FFM, fat free mass; MM, muscle mass; BCMI, body cellular mass index; ns, not significant.

**Table 10:** Other biological markers of CKD patients.

Biomarker	T0	T1	T0 vs. T1
**Platelet-to-lymphocyte ratio**	146.2 (86.2–398.7) ^a^	122.0 (54.7–337.5) ^a^	*p* = 0.004 ^b^
**Neutrophil-to-lymphocyte ratio**	1.9 (0.52–6.6) ^a^	1.3 (0.9–8.9) ^a^	ns ^b^
**Lymphocyte-to-monocyte ratio**	3.7 (1.6–10.2) ^a^	3.8 (1.6–8.1) ^a^	ns ^b^
**Lymphocyte (n/mm^3^)**	1.9 ± 0.7 ^c^	1.8 ± 0.6 ^c^	ns ^d^

^a^ Data expressed as a median and the minimum-maximum range is shown in brackets; ^b^ Applied test: Wilcoxon test; ^c^ Data expressed as mean ± standard deviation; ^d^ Applied test: *t*-test for paired data; Values of *p* ≤ 0.05 are considered statistically significant; Abbreviations: ns, not significant.

**Table 11:** Other biological markers of healthy subjects.

Biomarker	T0	T1	T0 vs. T1
**Platelet-to-lymphocyte ratio**	121.0 ± 27.4 ^c^	109.9 ± 27.7 ^c^	ns ^d^
**Neutrophil-to-lymphocyte ratio**	1.4 (0.5–3.2) ^a^	1.5 ± 0.4 ^c^	ns ^b^
**Lymphocyte-to-monocyte ratio**	4.6 ± 1.4 ^c^	5.1 ± 1.4 ^c^	ns ^d^
**Lymphocyte (n/mm^3^)**	2.3 ± 0.5 ^c^	2.4 ± 0.5 ^c^	ns ^d^

^a^ Data expressed as a median and the minimum–maximum range is shown in brackets; ^b^ Applied test: Wilcoxon test; ^c^ Data expressed as mean ± standard deviation; ^d^ Applied test: *t*-test for paired data; Values of *p* ≤ 0.05 are considered statistically significant; Abbreviations: ns, not significant.

**Table 12:** PREDIMED questionnaire.

Degree of Mediterranean Diet Adherence	CKD Patients			Healthy Subjects		
	T0	T1	*p* (McNemar’s Test)	T0	T1	*p* (McNemar’s Test)
**Minimal Adherence (%)**	0	0	ns	0	0	ns
**Average Adherence (%)**	86	81	ns	80	80	ns
**Maximal Adherence (%)**	14	19	ns	20	20	ns

Abbreviation: ns, not significant.

**Table 13:** IPAQ questionnaire.

Degree of Physical Activity	CKD Patients			Healthy Subjects		
	T0	T1	*p* (McNemar’s Test)	T0	T1	*p* (McNemar’s Test)
**Inactive (%)**	48	52	ns	60	60	ns
**Sufficiently Active (%)**	48	44	ns	40	40	ns
**Very Active (%)**	4	4	ns	0	0	ns

Abbreviation: ns, not significant.
